# Draft Genome Sequence of *Hydrogenibacillus schlegelii* MA48, a Deep-Branching Member of the *Bacilli* Class of *Firmicutes*

**DOI:** 10.1128/genomeA.00380-16

**Published:** 2017-01-19

**Authors:** Allison Maker, James Hemp, Laura A. Pace, Lewis M. Ward, Woodward W. Fischer

**Affiliations:** a Division of Geological and Planetary Sciences, California Institute of Technology, Pasadena, California, USA; b Department of Medicine, University of California, San Diego, La Jolla, California, USA

## Abstract

We report here the draft genome sequence of *Hydrogenibacillus schlegelii* MA48, a thermophilic facultative anaerobe that can oxidize hydrogen aerobically. *H. schlegelii* MA48 belongs to a deep-branching clade of the *Bacilli* class and provides important insight into the acquisition of aerobic respiration within the *Firmicutes* phylum.

## GENOME ANNOUNCEMENT

*Hydrogenibacillus schlegelii* MA48 was isolated from a eutrophic lake in Neufchatel, Switzerland (1). Closely related strains have also been found in volcanic soils in Antarctica (2) and various small pools and volcanic ash in Switzerland (3). *H. schlegelii*-related spores are widely distributed in cold environments; however, they appear to grow only in hot geothermal regions (3). *H. schlegelii* MA48 is thermophile (70°C [1, 2]) that grows under mildly acidic conditions (4.2 to 7.5 pH [4]). It is a facultative chemolithoautotroph that can couple hydrogen oxidation to aerobic respiration (5). It can also grow chemoorganoheterotrophically on numerous amino and organic acids. One study found *H. schlegelii* MA48 to be Gram positive (2), but others found it to be Gram variable (1, 4).

The genome of *Hydrogenibacillus schlegelii* MA48 (DSM 2000) was sequenced as part of a project to elucidate the evolutionary history of aerobic respiration within the *Firmicutes* phylum. Genome sequencing was performed at SeqMatic using the Illumina MiSeq sequencing platform. SPAdes 3.1.1 (6) was used to assemble the genome. The genome was screened for contaminants based on sequence coverage, G+C composition, and BLAST hits of conserved single-copy genes. Genome annotation was performed using the NCBI Prokaryotic Genome Annotation Pipeline. The draft genome is 2.61 Mb in size, assembled into 67 contigs. It contains 3,846 genes, 3,347 coding sequences (CDSs), two 16 small RNAs (sRNAs), 50 tRNAs, and three clustered regularly interspaced short palindromic repeat (CRISPR) arrays. It is estimated to be ~97% complete based on conserved single-copy genes (108/111).

Analysis of the genome identified numerous genes for aerobic respiration: complex I (NADH dehydrogenase), complex II (succinate dehydrogenase), complex III (cytochrome *bc* complex), an A-family heme-copper oxygen reductase, two B-family heme-copper oxygen reductases (7), a quinol *bd* oxidase, and a novel *bd* oxidase-related protein that receives electrons from cytochrome *c* instead of quinol (8, 9). Since *H. schlegelii* MA48 has a basal phylogenetic position within the *Bacilli* (4), these data help to constrain the relative timing of the acquisition of aerobic respiration within the *Firmicutes*. Genes for a Gram-positive type flagellum were also observed, suggesting the presence of only one membrane under certain physiological conditions.

### Accession number(s).

This whole-genome shotgun project has been deposited in DDBJ/EMBL/GenBank under the accession number JXBB00000000.
